# Recent progress on elucidating the molecular mechanism of plasmid-mediated colistin resistance and drug design

**DOI:** 10.1007/s10123-019-00112-1

**Published:** 2019-12-23

**Authors:** Jindan Kai, Sheng Wang

**Affiliations:** Department of Thoracic Surgery, Hubei Cancer Hospital, Wuhan, 430079 China

**Keywords:** Antibiotic resistance, Polymyxin, MCR-1, Drug design

## Abstract

Antibiotic resistance is a growing global challenge to public health. Polymyxin is considered to be the last-resort antibiotic against most gram-negative bacteria. Recently, discoveries of a plasmid-mediated, transferable mobilized polymyxin resistance gene (*mcr-1*) in many countries have heralded the increased threat of the imminent emergence of pan-drug-resistant super bacteria. MCR-1 is an inner membrane protein that enables bacteria to develop resistance to polymyxin by transferring phosphoethanolamine to lipid A. However, the mechanism associated with polymyxin resistance has yet to be elucidated, and few drugs exist to address this issue. Here, we review our current understanding regarding MCR-1 and small molecule inhibitors to provide a detailed enzymatic mechanism of MCR-1 and the associated implications for drug design.

**Electronic supplementary material:**

The online version of this article (10.1007/s10123-019-00112-1) contains supplementary material, which is available to authorized users.

## Introduction

**Fig. 1:**
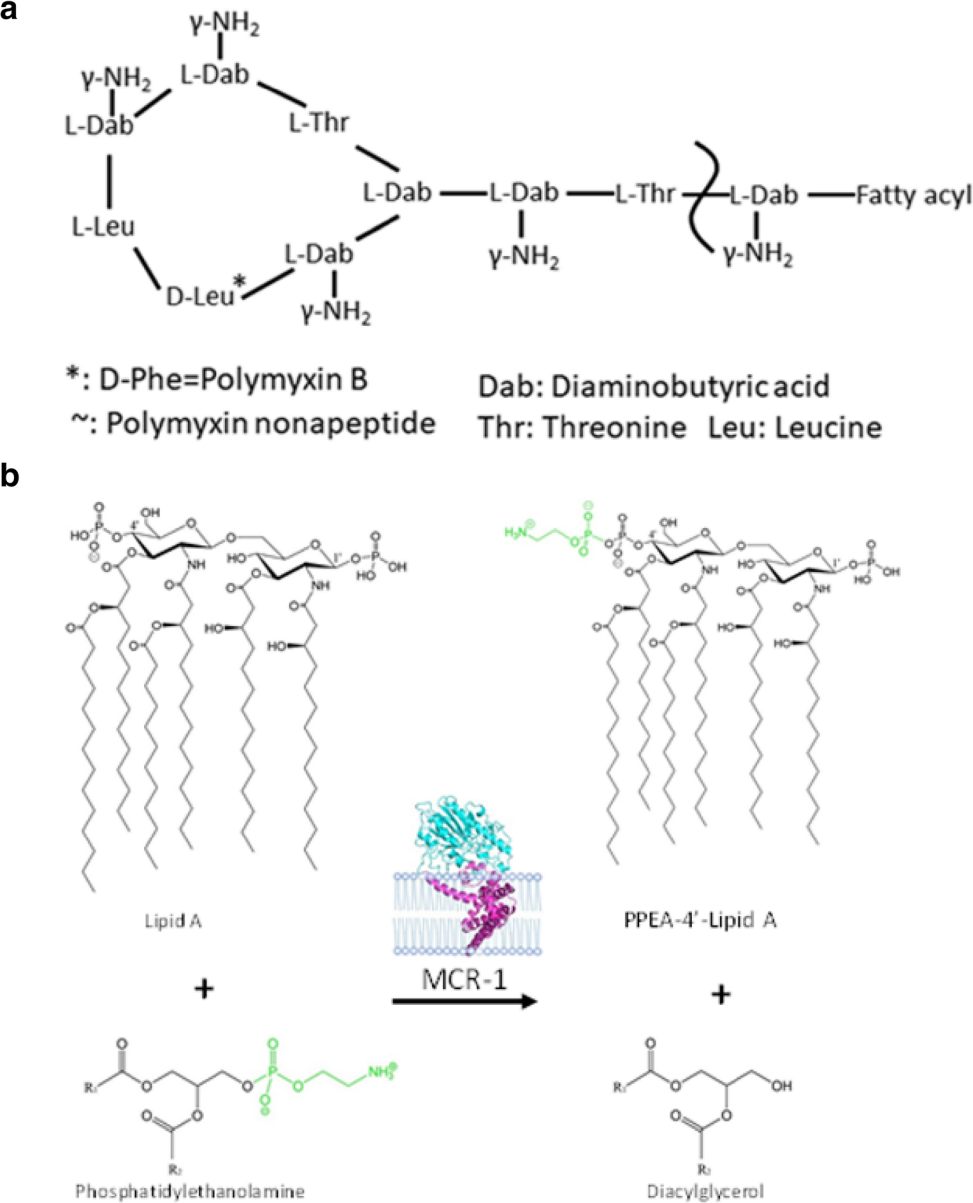
The structure of colistin and the PEA transfer reaction. **a** The structure of colistin. In polymyxin B, the d-Leu in colistin is replaced with d-Phe. **b** The transfer of phosphatidylethanolamine (PEA) to the 4′ and/or 1′ phosphates on lipid A is catalyzed by MCR-1

**Fig. 2:**
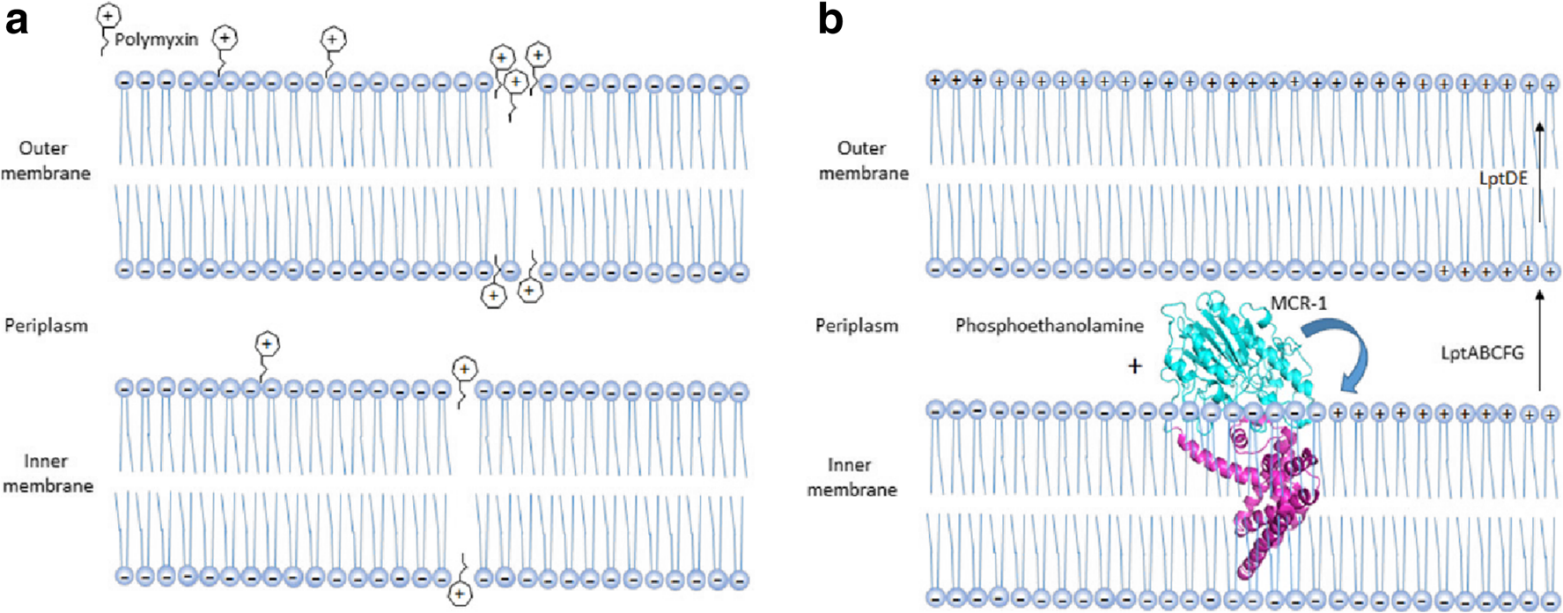
Polymyxin activity and working model and mechanism of MCR-1-induced polymyxin resistance. **a** Polymyxin interacts with the lipid A portion of gram-negative bacteria. The peptides cross the outer membrane and then interact with the cytoplasmic membrane to induce cytoplasmic membrane permeabilization and subsequent cell death. **b** The integral membrane protein MCR-1 normally localizes to the periplasmic side of the inner membrane and catalyzes the chemical modification of lipid A, yielding PEA-lipid A. The modified PEA-lipid A is then exported by LptABCFG and LptDE into the outer membrane, reducing the negative membrane charge and lowering the affinity of the bacterial surface to the cationic antibiotic polymyxin

Polymyxins are a cationic antimicrobial peptides discovered in 1947 (Ainsworth et al. 1947; Benedict and Langlykke 1947), of which there are five known types (polymyxin A–E) (Poirel et al. 2017). Polymyxins are polypeptides synthesized by *Bacillus*-like bacteria using nonribosomal peptide synthetases, and they play an important role in the innate immunity of gram-positive bacteria. For many years, polymyxin has been considered to be the last line of defense against multidrug-resistant bacteria in clinical treatment (Jeannot et al. 2017). Currently, there are only two types of polymyxins used in clinical settings, namely, polymyxin B and polymyxin E (colistin) (Rabanal and Cajal 2017). These polymyxins have similar decapeptide structures consisting of cyclic heptapeptide rings with tripeptide side chains acylated by fatty acids at the amino end (Fig. 1a) (Velkov et al. 2013). The only difference between polymyxins B and E is that d-phenylalanine in polymyxin B is replaced by d-leucine in colistin. Polymyxins also contain cationic l-α-γ-diamino-butyric acid residues that give them a positive charge of + 5 valence at pH 7.4 (Berglund et al. 2015; Trimble et al. 2016; Velkov et al. 2013). In addition, they also contain two hydrophobic components, d-phenylalanine and l-leucine at amino acid positions 6 and 7 in the fatty acid chain of polymyxin B, and d-leucine and l-leucine at positions 6 and 7 in colistin. The presence of these groups causes polymyxins to be amphiphilic, which is essential for their antimicrobial activity. It is generally believed that gram-negative bacteria are the target of polymyxins. The positively charged l-α-γ-diamino-butyric acid residue in polymyxins interact with the negatively charged phosphoric acid group of lipid A, replacing the calcium and magnesium ions previously united with the phosphoric group (Dixon and Chopra 1986). This action causes lipid A to become unstable and increases the permeability of bacterial membranes, leading to leakage of substances in bacteria and eventually bacterial death (Fig. 2b) (Falagas and Kasiakou 2005). Although lipid A is the initial target of polymyxins, the specific mechanism of polymyxins is unclear. Another antibacterial mechanism is endotoxin activity. The lipid A of lipopolysaccharide from gram-negative pathogens is an endotoxin, and polymyxins have the ability to bind and neutralize the lipopolysaccharide molecules released during cell lysis (Li et al. 2005). Another mechanism of action of polymyxins is to inhibit important respiratory enzymes in the bacterial inner membrane, i.e., type II NADH-quinone oxidoreductases (NDH-2) (Deris et al. 2014). Since the 1950s, polymyxin has been used in medical clinics and in aquaculture. However, due to its nephrotoxicity and neurotoxicity, as well as the development and use of a new generation of broad-spectrum antibiotics, it has not been widely used clinically. In the late 1990s, polymyxin was reapplied for clinical use due to the emergence of gram-negative bacteria with multiple drug resistance, especially carbapenem-resistant *Enterobacter*, and is considered to be the last line of defense against bacterial infection. Thus, the mechanism of its drug resistance has subsequently received extensive international attention (Elwood et al. 1966; Koch-Weser et al. 1970; Nord and Hoeprich 1964).

Phosphoethanolamine (PEA) transferase catalyzes the addition of a PEA group to the 1′ or 4′ phosphate group of lipid A to produce PEA-lipid A (Gao et al. 2016; Liu et al. 2016). Previous reports have shown that the chromosomally encoded proteins EptA (the *Neisserial* lipooligosaccharide PEA Transferase A), EptC (the flagellar rod protein), and other phosphoethanolamine transferases can complete the transfer of ethanolamine phosphate (Anandan et al. 2017; Fage et al. 2014; Wanty et al. 2013). The PEA group is supplied by phosphatidylethanolamine, which is ubiquitous in the inner membranes of gram-negative bacteria, and the reaction is catalyzed by proteins on the periplasmic surface of the inner membrane. However, the specific mechanism associated with this reaction remains unclear (Poirel et al. 2017). In 2013, Vrielink et al. determined the catalytic domain structure of the chromosomally encoded polymyxin resistance protein EptA (Wanty et al. 2013). The following year, Trent et al. determined the catalytic domain structure of EptC, another chromosomally encoded polymyxin resistance protein (Fage et al. 2014). A major breakthrough in this field occurred when Vrielink et al. obtained the crystal structure of full-length EptA (Anandan et al. 2017), which showed that EptA contains 5 transmembrane alpha helices that aid in the insertion of EptA into the membrane. At the same time, several additional helices in EptA connect the catalytic and transmembrane domains. Recently, a new member of the PEA transferase family of proteins, mobile colistin resistance (MCR), was identified (Liu et al. 2016). MCR is a plasmid-encoded polymyxin resistance protein that can add phosphoethanolamine to the phosphoric group of lipid A (Fig. 1b), resulting in more positive charges on lipopolysaccharides and low/decreased affinity of bacterial surfaces to the cationic antibiotic polymyxin (Fig. 2b) (Sun et al. 2017; Xu et al. 2018b). As polymyxins are regarded to be the last line of defense against bacterial infections, they have attracted increased worldwide attention after MCR-1 was first reported in late 2015 (Arcilla et al. 2016; Hu et al. 2016b; Mulvey et al. 2016; Rawson et al. 2016; Rolain and Olaitan 2016; Ruppe et al. 2016; Tse and Yuen 2016; Zhang et al. 2016; Zhi et al. 2016). At present, researchers have reported eight additional different MCR family members (MCR-2 through MCR-9) (AbuOun et al. 2018; Borowiak et al. 2017; Carattoli et al. 2017; Carroll et al. 2019; Wang et al. 2018; Xavier et al. 2016; Yang et al. 2018; Yin et al. 2017). Protein sequence analysis suggests that the nine members of the MCR family of proteins have a highly identical secondary structure, with a high degree of conservation observed for many amino acid residues, especially those in the active site and PE-interacting cavities (Supplementary Fig. 1). For example, residues E246, T285, H390, D465, and H466 in the zinc-binding cavity and residues N108, T112, E116, S330, K333, H395, and H478 in the PE-binding cavity in MCR-1 are also located in similar positions in the other 8 MCR members (Zhang et al. 2019a). These observations constitute a structural paradigm for understanding the mechanism of action for MCR family proteins, informing the design of inhibitors to bypass colistin resistance. In addition, the results of many evolutionary, genomic, and mechanistic studies have suggested that MCR members are functional, unified, and equivalent (Sun et al. 2017; Zhang et al. 2019a; Zhang et al. 2019b; Zhang et al. 2019c). Because the MCR-1-encoding gene appears to be the most prevalent protein having been detected in nearly 50 countries across 6 continents, as well also the most studied, we primarily focused on MCR-1 in the following discussion (Falgenhauer et al. 2016; McGann et al. 2016; Poirel et al. 2017; Schwarz and Johnson 2016; Sun et al. 2018). The expression of MCR-1 in *E. coli* can increase the minimum inhibitory concentration (MIC) of polymyxins by 4–8-fold (Hinchliffe et al. 2017; Hu et al. 2016a; Stojanoski et al. 2016; Wei et al. 2018; Xu et al. 2018a). Therefore, MCR-1 alone is sufficient to confer polymyxin resistance to *E. coli* and other intestinal bacteria without the aid of other resistance mechanisms. In addition to conferring resistance to polymyxin antibiotics, studies have shown that MCR-1 can also confer bacterial resistance toward lysozyme (Sherman et al. 2016). Since Stojanoski et al. first reported the crystal structure of the catalytic domain of MCR-1, the key catalytic sites of MCR-1 have been subsequently elucidated, but the substrate-binding sites of MCR-1 have yet to be identified, which is crucial for understanding the complete reaction catalyzed by this protein and for designing corresponding inhibitors. Recently, our group identified two promising substrate analogs, one of which can inhibit the polymyxin resistance conferred by MCR-1 (Wei et al. 2018). In this review, we will discuss recent structural research advances in plasmid-mediated colistin resistance protein from studies by our lab and others and examine important features associated with this family of proteins to promote a better understanding of the related molecular mechanism and drug design for plasmid-mediated colistin resistance.

## Overall structure of cMCR-1

**Fig. 3:**
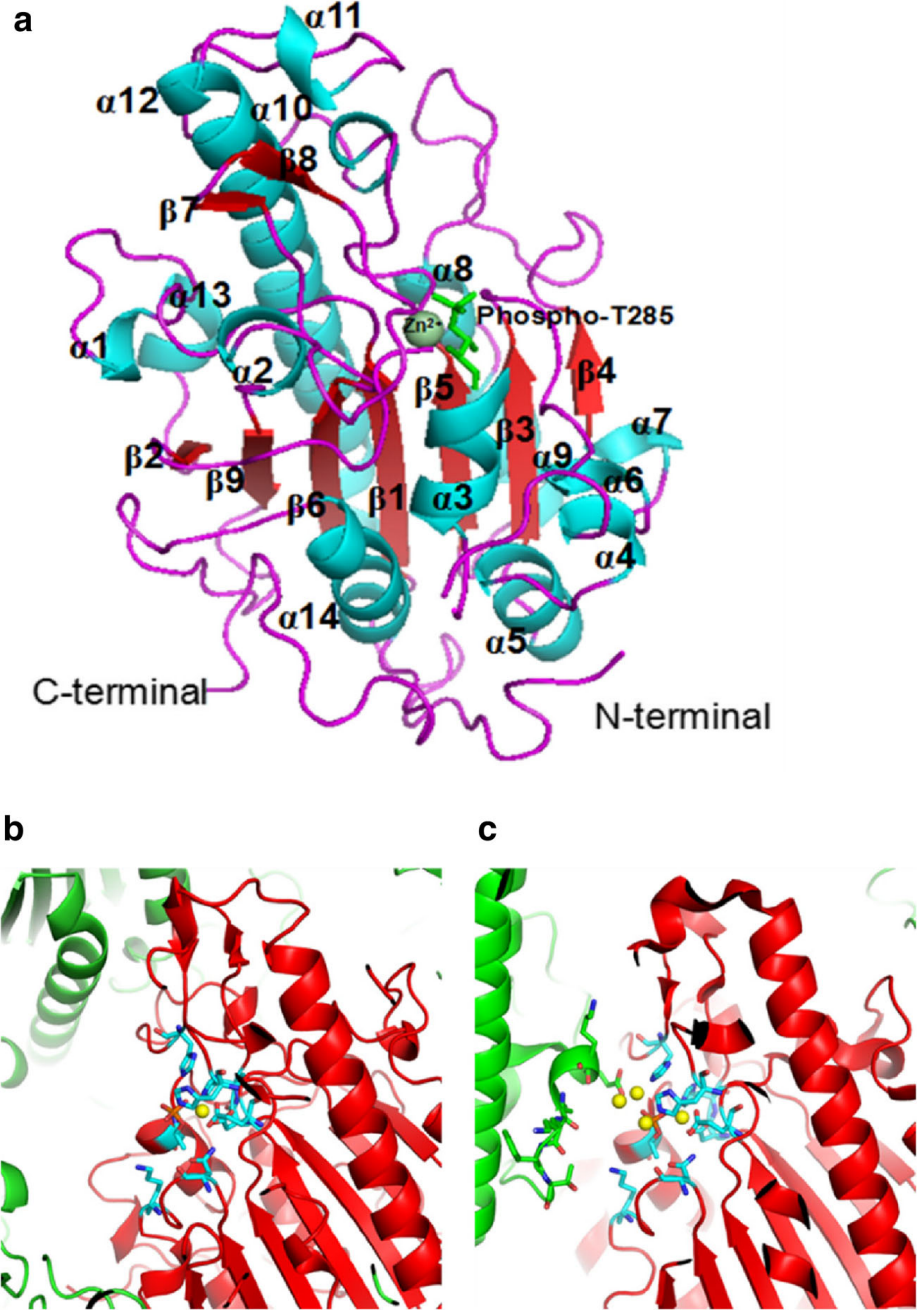
Structural characteristics of cMCR-1. **a** Structure of the C-terminal catalytic domain of MCR-1 (cMCR-1). The α-helices (blue), β-strands (red), loops (purple), phospho-T285 (light green), and the N- and C-termini are labeled. Zn^2+^ (dark green) is displayed as a sphere. **b** Packing diagram showing mono-zinc occupied MCR-1 in space groups P2_1_2_1_2_1_. **c** Packing diagram showing multi-zinc occupied MCR-1 in space groups P4_3_2_1_2. The zinc ion is shown as a yellow sphere, and the catalytic core residues are presented as stick-model form (cyan). The focused MCR-1 molecule is shown in red, while the other MCR-1 molecules are shown in green

Stojanoski et al. first reported the crystal structure of the catalytic domain of MCR-1 (cMCR-1), revealing that its active site has similar structure to other phosphoethanolamine transferases (Stojanoski et al. 2016). Subsequently, three different groups reported almost the same crystal structure for the MCR-1 catalytic domain that was reported by Stojanoski et al. (Hinchliffe et al. 2017; Hu et al. 2016a; Ma et al. 2016). Subsequently, the crystal structure of the MCR-2 catalytic domain, exhibiting a high similarity to the MCR-1 sequence, was also analyzed (Coates et al. 2017). The results showed that the catalytic domain of MCR-2 has a very similar structure to that of MCR-1 (Fig. 3) (Coates et al. 2017). Threonine 285 (T285) was identified as a conserved nucleophilic attack group due to it being phosphorylated in cMCR-1, and this residue is conserved among the nine MCR family members. The structure of cMCR-1 shows an α/β/α fold in which the central β-sheets are enveloped by α helices (Fig. 3a). There are 9 β-sheets in this fold, 7 of which are located in the middle, while the remaining two short β-sheets are located off to one side, away from the center. Surrounding the central β-sheets are 14 α-helices. This α/β/α fold is common among proteins in the alkaline phosphatase superfamily (pfam00245), allowing for the cleavage of ester bonds such as phosphoester linkages, an activity that is likely required for MCR-1 to transfer PEA to lipid A (Mohamed and Hollfelder 2013).

## Phosphorylated Thr285 and Zn^2+^-dependent catalytic core

**Fig. 4:**
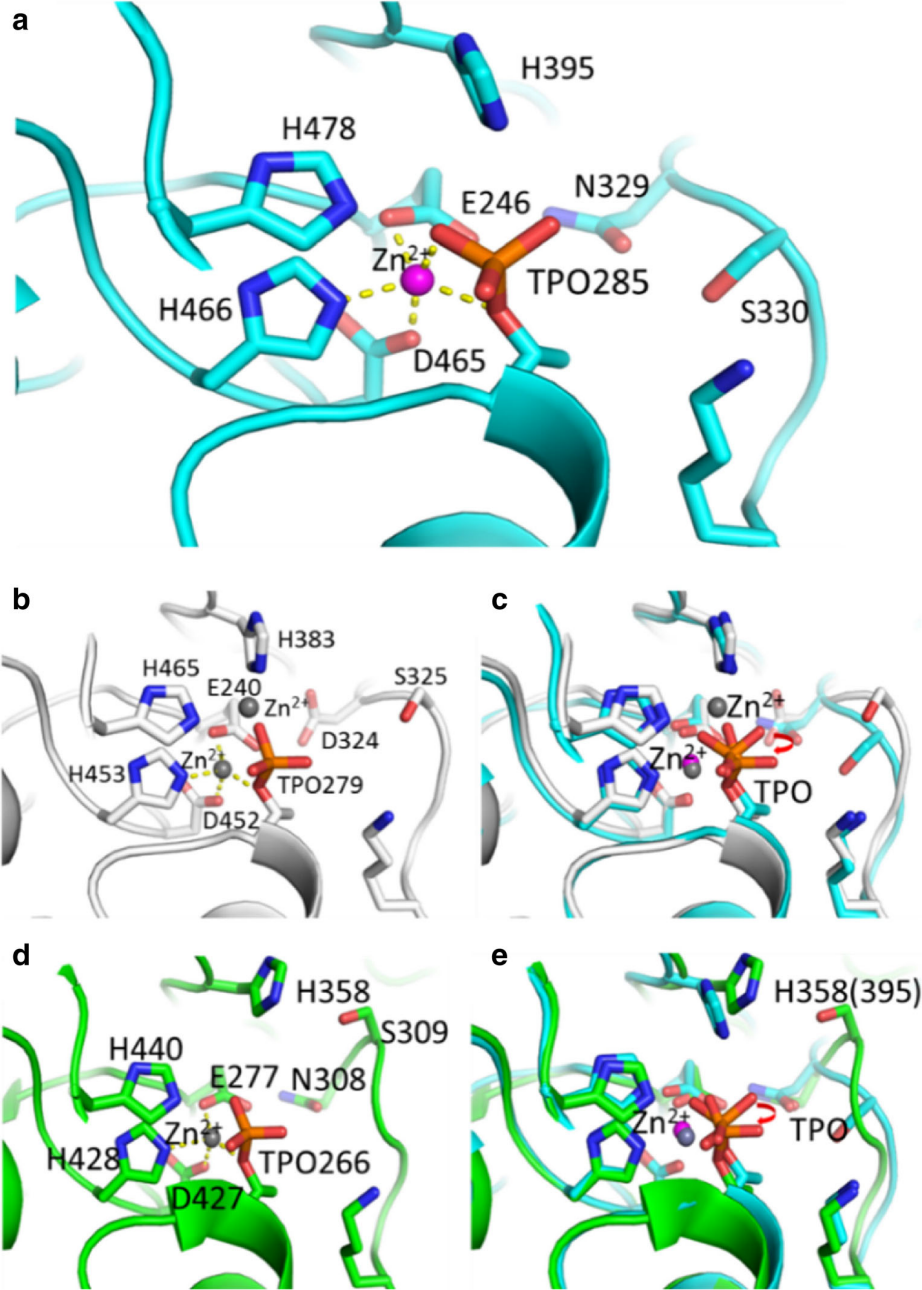
High conservation of a catalytic core in cMCR-1, cEptA, and cEptC. **a** Residues around the Zn^2+^ ion in cMCR-1. The Zn^2+^ ion is penta-coordinated, as indicated by yellow dotted lines. Glu246, Asp465, and His466 as well as the hydroxyl oxygen atom of Thr285 and one of the phosphate oxygen atoms are involved in the coordination of Zn^2+^. **b** Residues around the Zn^2+^ ion in cEptA. The coordination of two Zn^2+^ ions is shown by the yellow dotted line. One oxygen atom in the phosphate group swings aside to coordinate another Zn^2+^ ion together with H383 and H465. **c** Superposition of cMCR-1 and cEptA. The oxygen atoms in the phosphate group are shown in different positions by rotating at a specific angle. **d** Residues around the Zn^2+^ ion in cEptC. The coordination of Zn^2+^ is shown by the yellow dotted line. One oxygen atom in the phosphate group swings aside but does not coordinate the Zn^2+^ ion, unlike in cMCR-1. **e** Superposition of cMCR-1 and cEptC. The oxygen atoms in the phosphate group are shown in different positions by rotating at a specific angle. Subsequently, the side chain of H358 in cEptC (labeled as H395 in cMCR-1 in the parentheses) swings aside

In a sequence alignment of MCR-1 with two other members of the PEA transferase family (EptA and EptC), a conserved Thr285, which was suggested to be the active-site nucleophile in EptA and EptC, can be observed (Fig. 4b, d). The conserved putative nucleophile Thr285 in cMCR-1 resides at the N-terminus of the α3 helix, which is identical to that observed in the catalytic domain of EptA (cEptA) and EptC (cEptC) (Fig. 4a, b, d). Thus, the helical dipole may help to stabilize a nucleophilic alkoxide form of the side chain. A phosphoryl group is also clearly observed from the electron density map and is attached to Thr285 to form phosphothreonine, mimicking a PEA-enzyme intermediate (Fig. 4a). In addition, the mutation of Thr285 to alanine, which abolishes the potential for phosphorylation at this site, leads to an impairment of colistin resistance.

A metal ion is present adjacent to Thr285, even though no metal ion was purposely added throughout the crystallization process. This metal ion was also observed in cEptA and cEptC (Fig. 4a, b, d). The metal ions in cEptC, cEptA, and cMCR-1 were determined to be Zn^2+^ by anomalous diffraction, X-ray fluorescence excitation scan, or atomic absorption spectroscopy assay (Fage et al. 2014; Wanty et al. 2013; Wei et al. 2018).

The Zn^2+^ ion is penta-coordinated by Glu246, Asp465, and His466, as well as the hydroxyl oxygen atom of Thr285 and one of the phosphate oxygen atoms within the distance of 2.0 to 2.3 Å (Fig. 4a). Zn^2+^ was proposed to stabilize the nucleophile Thr285 that is crucial for MCR-1 activity. The residues involved in coordinating Zn^2+^ are highly conserved among PEA transferase family members and are also believed to be crucial for the function of MCR-1. Mutating a number of these residues to alanine greatly decreased the colistin resistance of *mcr-1*-transduced bacteria. These results strongly suggest that the Zn^2+^ ion, together with phosphorylated Thr285 and the surrounding residues, forms the active site of MCR-1.

## cMCR-1 in complex with substrate analogs and other small molecules

At present, several research groups, including our own, have analyzed the crystal structure of cMCR-1. However, the substrate-binding site, the most important site needed to understand the mechanism of MCR-1 activity, has not been reported. Therefore, making the acquisition of the structure of MCR-1 and its substrate is of great importance. Using a series of crystal soaking steps, we tested dozens of different conditions that ultimately resulted in the acquisition of high-resolution crystal structures of cMCR-1 with two substrate analogs, ethanolamine (ETA, substrate phosphatidylethanolamine analog) and d-glucose (substrate lipid A analog).

First, the structure of cMCR-1 combined with ethanolamine was analyzed. From the electron density, ethanolamine could be clearly observed next to phosphorylated T285. Ethanolamine was primarily observed to interact with N329 and phosphorylated T285. When cMCR-1 was bound with ethanolamine, the lateral chain of H395 appeared to rotate approximately 50°. Further analysis of the water molecules in the catalytic region of cMCR-1 before and after the binding of ethanolamine revealed that network of water molecules in the catalytic region is greatly altered after the binding of ethanolamine and that K333 is very important for the formation of this network of water molecules. Simultaneously, we obtained the structure of cMCR-1 complexed with d-glucose, which clearly showed the presence of d-glucose in the semi-open pocket formed by T283, S284, T285, Y287, P481, and N482. Moreover, the electron density also showed that d-glucose had a clear density. Interestingly, other molecules, such as d-xylose, glycerol, and d-sorbitol, have been reported to bind to the same semi-open pocket, indicating that this pocket has a substrate specificity (Liu et al. 2018; Ma et al. 2016; Stojanoski et al. 2016).

## Multiple zinc positions and possible multiple states in the catalytic region of MCR-1

A mono-zinc site (Zn1) was unambiguously identified in our previously reported structure (Fig. 3a, b) (Wei et al. 2018) that is also supported by the structure of mono-zinc cMCR-1 in the P21 space group (PDB: 5LRN). However, di-zinc (PDB: 5LRM) and 4-zinc (PDB: 5K4P) structures were also observed by researchers (Fig. 3c). This complex zinc stoichiometry could result from putative multiple states in the catalytic region of cMCR-1, the presence of which have been indicated from the previously noted conformational change in H395 when the substrate analog ethanolamine binds near T285. To obtain additional insights into the mechanism associated with multiple states of the catalytic region, MCR-1 homologs are also currently under careful analysis.

The closest homologs to MCR-1 are two members of the PEA transferase family, the lipopolysaccharide export system protein EptA from *Neisseria meningitidis* and the phosphoethanolamine transferase EptC from *Campylobacter jejuni*. When the catalytic center of these three homologs (cMCR-1, cEptA, and cEptC) is compared (Fig. 4c, e), a notable and novel rotation of the phosphate group of the crucial phosphorylated threonine and a rotation of H395 (equivalent to H383 in cEptC and H358 of cEptA) are observed, suggesting the existence of different states of the catalytic region.

In cMCR-1, one of the oxygen atoms in the phosphate group of the phosphorylated threonine is the fifth ligand to coordinate the native Zn^2+^ ion (Fig. 4a). However, in the reported structure of cEptA, the phosphate group of the phosphorylated threonine is rotated, and the equivalent fifth ligand appears swung aside to leave this native Zn^2+^ to be tetrahedrally coordinated. This fifth ligand turns to coordinate another Zn^2+^ ion together with H383 and H465 of cEptA (Fig. 4b, c). In the reported structure of cEptC, this fifth ligand also swings aside, leaving the native Zn^2+^ ion to be tetrahedrally coordinated. However, H358 of cEptC (equivalent to H383 of cEptA and H395 of cMCR-1) shifts away and is no longer in a position where it can coordinate the second Zn^2+^ ion (Fig. 4d, e). As a result, the second Zn^2+^ ion is not observed in the reported structure of cEptC. As MCR-1, EptA, and EptC can all modify lipid A with PEA, and because the components of the catalytic region are so conserved, the different conformations in the catalytic region of cMCR-1, cEptA, and cEptC may be due to different reaction steps other than distinct catalytic mechanisms.

In addition, as noted above, di- and multi-zinc crystal structures of MCR-1 have been observed in recent studies. Although some Zn^2+^ ions are artificially introduced by crystal packing, the Zn^2+^ ion coordinated by T285 has repeatedly been observed in many structural studies and has been shown to be very important through T285 mutational analyses. Another Zn^2+^ ion has been shown to be coordinated by H395 in the cEptA structure and in some reported cMCR-1 structures. However, this phenomenon has not been observed in other reported cMCR-1 structures or in the cEptC structure, possibly due to the involvement of different catalytic states of cMCR-1 by this Zn^2+^, as mentioned above. The Zn^2+^ ion coordinated by H395 has also been shown to be crucial for colistin resistance from both in vitro and in vivo mutational assays of nearby residues. Thus, these two Zn^2+^ ion-coordinating residues (H395 contacting Zn^2+^ and T285 contacting Zn^2+^) could both be very important and be involved in multiple states of the entire enzymatic process of MCR-1. The results of these studies have yielded clues that stepwise multiple states occur in the catalytic region of MCR-1.

## Different dynamic disulfide bond states in pairs of cysteine residues in MCR-1

**Fig. 5:**
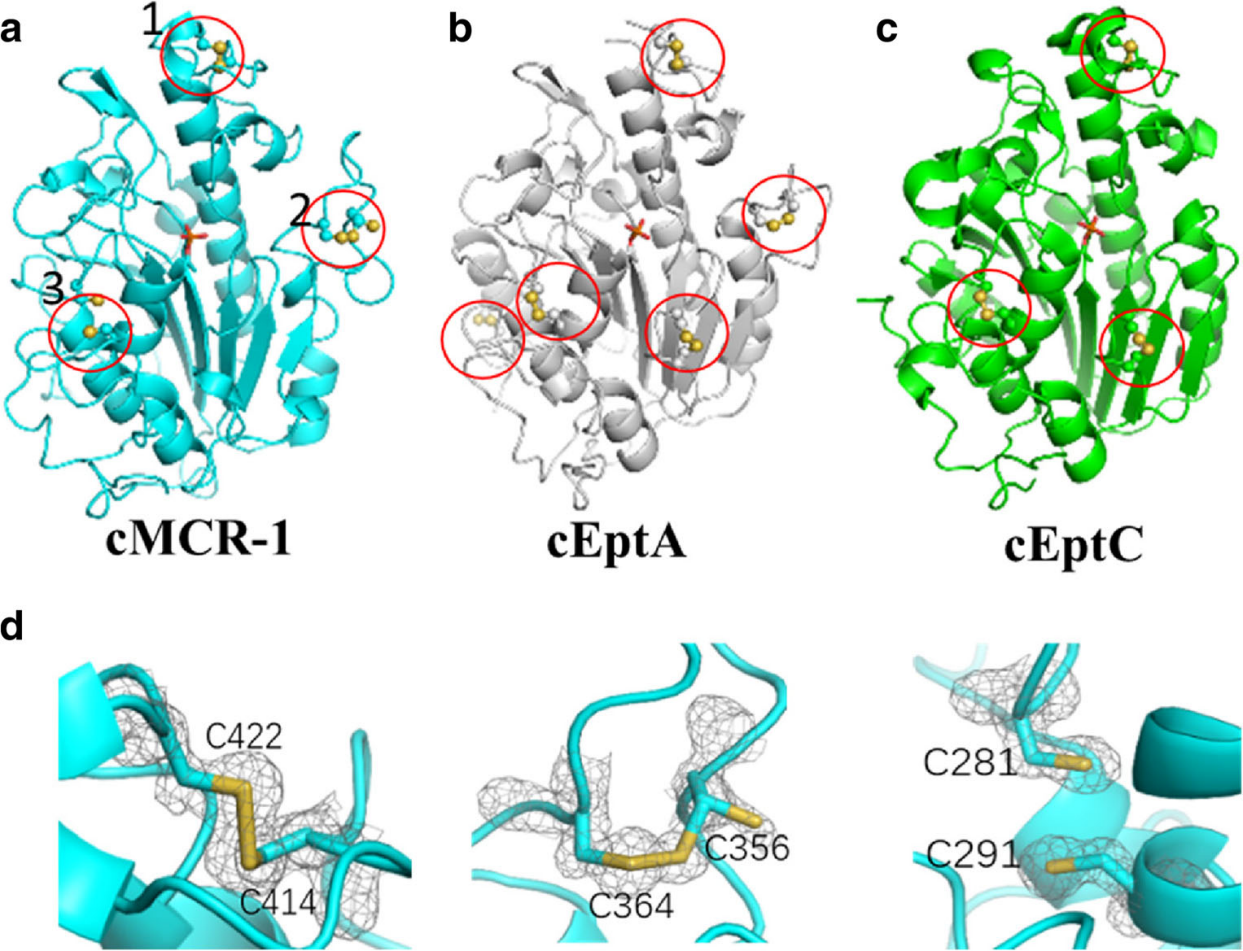
Multiple pairs of cysteine residues are present in cMCR-1, cEptA, and cEptC. **a**–**c** Multiple pairs of cysteine residues are common features in phosphoethanolamine transferase family proteins, including cMCR-1, cEptA, and cEptC, which have 3, 5, and 3 pairs, respectively. Disulfides (yellow) are highlighted by red circles. **d** In cMCR-1, the 3 pairs of cysteine residues exhibit 3 different oxidation states, where all, some, or none are oxidized. Cys281 and Cys291 are not observed in their oxidized form. Cys356 and Cys364 are observed in partially oxidized forms, while Cys414 and Cys422 are observed in their fully oxidized form. Different states of disulfide bond formation are also observed in cEptA

There are 3 pairs of disulfide bonds surrounding the phosphorylated T285 (Fig. 5a). Multiple pairs of cysteines are common features in PEA transferase family proteins, including EptA and EptC, which have 5 pairs and 3 pairs, respectively (Fig. 5b, c) (Fage et al. 2014; Wanty et al. 2013). Compared with the cysteine residues in EptC, the 3 pairs observed in MCR-1 exhibit a different pattern, with the absence of C312-C316 in EptC and the existence of C364-C356 in an extra loop of MCR-1. Additionally, C356-C364 and C414-C422 are conserved among MCR-1, EptA, and EptC.

It is interesting that the 3 pairs of cysteine residues in cMCR-1 display 3 different disulfide bond states, which was observed in our work, from the fully, partially (in the same pair of two cysteines, one cysteine has much less electron density, showing partially occupancy, compared with the other cysteine), to none being oxidized (Fig. 5d) (unpublished data). From the crystallographic results, C414-C422 has been observed to be fully oxidized and form a disulfide bond, and C356-C364 is observed in a partially oxidized form, while C281-C291 is not in an oxidized form. Different states of cysteine residues pairs are also present in cEptA, suggesting might be an important feature in PEA transferase family. Whether these different states with respect to the pairs of cysteine residues are dynamic and can be changed was also tested. The cMCR-1 crystal was soaked in H_2_O_2_ before diffraction data collection and structure determination. The results showed that the partially and unoxidized forms of cysteine residues became fully oxidized disulfide bonds, and no obvious structural changes were detected in other regions of cMCR-1, suggesting that some disulfide bonds in cMCR-1 could be dynamic oxidized environment in vitro. In addition, the results of a bacterial growth assay confirmed the importance of these pairs of cysteines, as mutations of these pairs bacteria growth under polymyxin stress.

## Full-length MCR-1

With respect to MCR-1, only the crystal structure of cMCR-1 has been obtained to date, and an understanding of the structural characteristics of full-length MCR-1 is urgently needed to further elucidate the catalytic mechanism of MCR-1.

First, the relative orientation of the catalytic and membrane domain was determined with the identification of the Zn^2+^-dependent catalytic core, two putative substrate-binding pockets, and three important pairs of cysteines. The two putative binding pockets and phosphorylated T285 are on the same surface side as cMCR-1, leading to the conclusion that this surface side faces down toward the inner membrane. Thus, lipid A and phosphatidylethanolamine can bind to their respective binding pockets and be ready for the reaction with the aid of the transmembrane domain of MCR-1 and three pairs of cysteines. The nucleophile T285 would then be involved in transferring PEA to nearby lipid A.

As previously mentioned, EptA and EptC are the most similar proteins to MCR-1. Sequence alignments show that the similarity between the MCR-1 and EptA or EptC sequences is approximately 35%. This level of similarity is common among different proteins within a large family, and the folding of the catalytic domain of EptA or EptC was similar to that of the catalytic domain of cMCR-1 as well. More information could be extracted from the knowledge of the full-length EptA or EptC proteins. Fortunately, a research group recently solved the crystal structure of full-length EptA (Anandan et al. 2017), which was then used as a homologous template to model the full-length MCR-1. The simulation results showed that MCR-1 and EptA have a highly similar structure. The homologous modeling of the full-length MCR-1 allowed for the transmembrane domain of the full-length MCR-1 protein to be analyzed in detail. The structure shows that MCR-1 contains 5 transmembrane alpha helices as well as several helices that connect the catalytic and transmembrane domains. Further analysis of the amino acid composition in the transmembrane region shows that there are many positively charged R/K amino acids in the transmembrane region. Thus, the transmembrane domain of MCR-1 has a concentrated positive potential, while the catalytic domain has a cross-distribution of positive and negative potential. This feature also sets up the foundation for the interaction between the transmembrane region, which has a positive potential, and the negatively charged lipid A.

## Implications for drug design

Currently, multiple approaches are under development for the treatment of antibiotic-resistant superbugs, including polymyxin-resistant bacteria (Daly et al. 2017). To the best of our knowledge, there are three primary approaches being investigated to reduce MCR-1-associated colistin resistance. The first solution is the development of novel antibiotics against MCR-positive organisms, such as eravacycline (Fyfe et al. 2016), plazomicin (Denervaud-Tendon et al. 2017), and artilysin (Schirmeier et al. 2018). Another method appears to be the mainstream approach involving the effective administration of colistin as well as the potential use of combination therapies with additional agents to generate synergistic associations. These agents can include antibiotics that are typically restricted for use against gram-positive bacteria, such as amikacin (Bulman et al. 2017; Zhou et al. 2017), aztreonam (Bulman et al. 2017), rifampin (Brennan-Krohn et al. 2018; Li et al. 2018), azithromycin (Brennan-Krohn et al. 2018; Li et al. 2018), clarithromycin (MacNair et al. 2018), linezolid (Brennan-Krohn et al. 2018), azidothymidine (Hu et al. 2019), and derivatives of tryptamine (Barker et al. 2019). Alternatively, natural products acting as adjuvants can be used, some of which can interact with lipopolysaccharides to perturb the outer bacterial membrane, such as pentamidine (Stokes et al. 2017) and meridianin D analogs (Huggins et al. 2018). In contrast, other adjuvants do not have specific roles insofar as we know, such as resveratrol (Cannatelli et al. 2018), pterostilbene (Zhou et al. 2018), osthole (Zhou et al. 2019), and niclosamide (Domalaon et al. 2019). The last but most important direction is to identify specific drugs targeting MCR. Several approaches have been reported to reduce MCR expression at the gene level, such as the use of peptide-conjugated phosphorodiamidate morpholino oligomers (PPMOs) to target *mcr-1* mRNA (Daly et al. 2017), peptide nucleic acid against the *mcr-1* gene (Nezhadi et al. 2019), and the CRISPR/Cas9 system to target *mcr-1*-harboring plasmids (Dong et al. 2019). However, few studies have investigated specific drugs targeting MCR, with promising results only having been observed for 1-phenyl-2-(phenylamino) ethanone derivatives (Lan et al. 2019) and the lipid A analog ethanolamine (Wei et al. 2018), both of which bind the cavity pocket. With the identification of the Zn^2+^-dependent catalytic core and two putative substrate-binding pockets, the use of targeted drug design has become a highly promising approach, especially with the detailed information of how substrate analogs and other small molecules bind to cMCR-1 (Son et al. 2018).

First, whether the Zn^2+^-dependent catalytic core and two putative substrate-binding pockets are important enough as drug target sites was investigated. The importance of the Zn^2+^-dependent catalytic core has been repeatedly confirmed by our lab and others through MIC measurements of strains carrying MCR mutations in residues around the catalytic core. Among these mutations, T285 has been shown to be particularly important, as MCR activity was almost completely abolished when T285 was mutated to alanine. To determine whether the substrate-binding site plays an important role in the activity of MCR-1, the mutation of residues around these sites has also been tested. The full-length membrane protein MCR-1 was extracted using an appropriate concentration of the detergent *n*-dodecyl-beta-d-maltoside (DDM) to maintaining its activity. Because both substrates of MCR-1 are water-insoluble substances, it is difficult to detect this enzyme activity in vitro, which is also a bottleneck in this field. Recently, researchers have used a fluorescently labeled substrate, 1-acyl-2-{12-([7-nitro-2-1,3-benzoxadiazol-4-yl) amino]dodecanoyl}-sn-glycero-3-phosphoethanolamine (NBD-PEA), to detect the semi-enzymatic activity of MCR-1 (Anandan et al. 2017). This assay was also used in mutation analyses of potentially important amino acids involved in binding phosphatidylethanolamine (ethanolamine-bound sites). The results showed that MCR-1 harboring amino acid mutations around the ethanolamine-binding pocket could not the substrate NBD-PEA. Since the substrate analog ethanolamine likely occupies the binding pocket of the substrate phosphatidylethanolamine, the addition of ethanolamine could block the enzyme activity of MCR. Therefore, the activity of this enzyme was assessed after adding ethanolamine, the results of which showed that 10 mM ethanolamine could completely block the enzymatic reaction.

An effort has also been made to determine the affinity of substrate analogs toward MCR-1 in vitro. For instance, the microscale thermophoresis (MST) method was used to determine the affinity of ethanolamine toward cMCR-1, with the results showing that ethanolamine could only bind cMCR-1 at a low affinity of *Kd* = 605 + 93.3 μM. These results indicate that further modification of the ethanolamine structure is needed to improve its affinity to this protein.

**Fig. 6:**
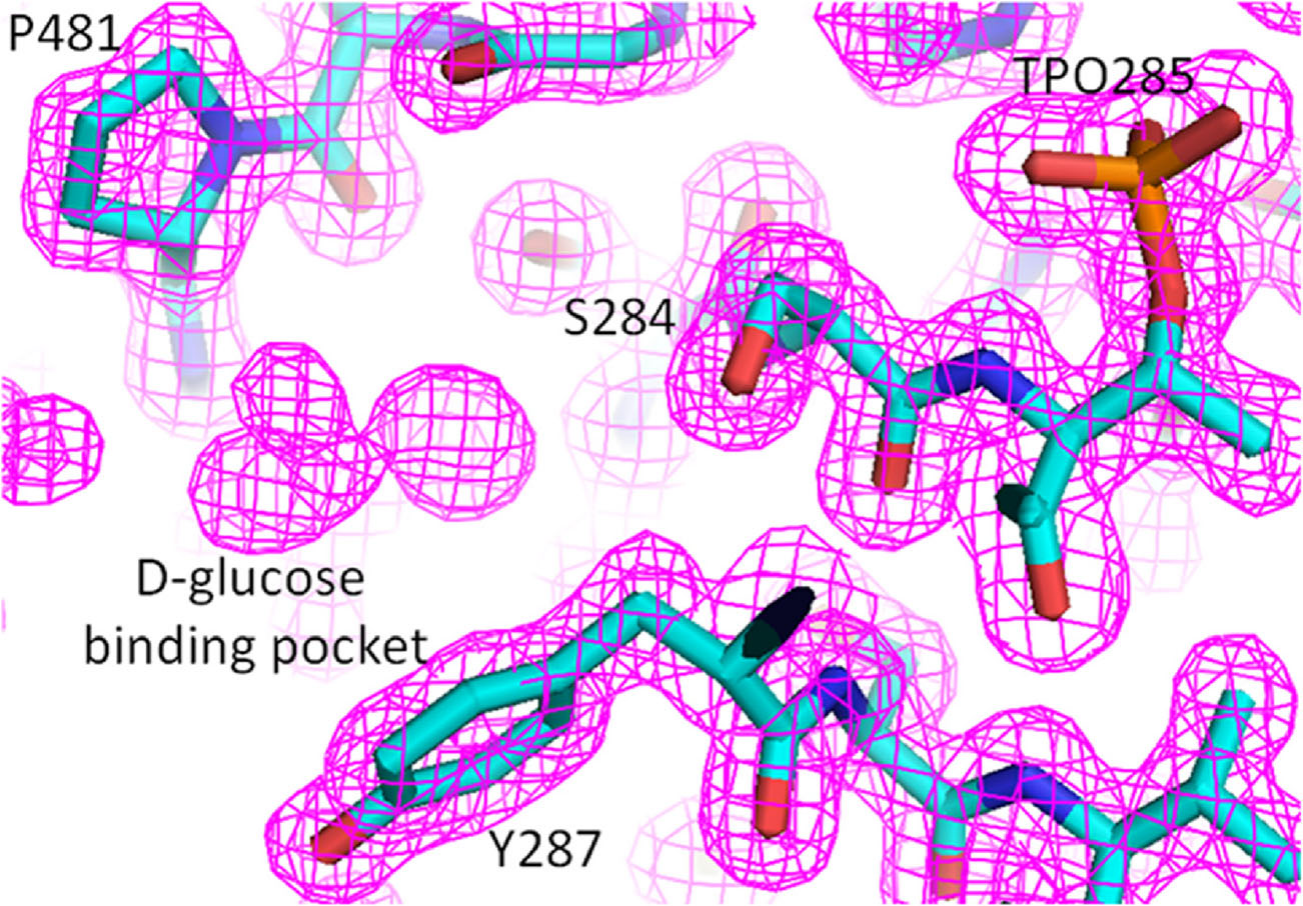
Unknown electron density in the d-glucose-binding pocket. Specific small molecules in the d-glucose-binding pocket in the 2Fo-Fc electron density map. The map is contoured at a 1.0 σ level. Blue, nitrogen; red, oxygen

Attempts have also been made to further determine the affinity of d-glucose toward cMCR-1, but after many experiments, no adequate results have been obtained. Therefore, a careful re-evaluation of the cMCR-1 crystal structure data was performed. The results showed that there was a clear electron density at the d-glucose-binding pocket in the high-resolution structure of cMCR-1 in the Apo form without soaking the crystals in any small molecules, and none of the small molecules in the crystallization solutions could fit the electron density map (Fig. 6). Despite the little that is known about the origin of the observed electron density, these results demonstrated that the pocket may have become occupied by a substance in bacteria prior to protein crystallization. Interestingly, only when a high concentration of d-glucose was used in the crystal soaking assay was the unknown substance in the pocket removed and replaced by d-glucose.

To further confirm that ethanolamine can be used as an inhibitor of MCR-1 activity, the inhibitory effect of ethanolamine toward bacteria containing MCR-1 was also tested in vivo. The results clearly showed that ethanolamine could inhibit the expression of MCR-1 in a concentration-dependent manner under in the presence of 4 mg/mL polymyxin B, further confirming that ethanolamine can be used as an inhibitor of MCR-1 activity. Considering the structural paradigm and functional unification within MCR family, we assume that ethanolamine would also act as inhibitor of other MCR members, although this would require verification through further experimentation. Since another MCR substrate analog is d-glucose, which can be used as an energy source by bacteria, it was unclear whether it could inhibit MCR-1 activity in vivo. Thus, an inhibition assay using d-glucose was also performed, the results of which showed that it could not inhibit the growth of bacteria encoding MCR-1.

## Conclusion

In summary, most of the studies performed to date have focused on the detection of the *mcr-1* gene and assessing the characteristics of the plasmid-carrying bacteria, with only a few studies having investigated the structure and molecular mechanism of MCR-1. Due to the important potential hazard of *mcr-1*, studies on the mechanism of action and drug design for MCR-1 are urgently needed. Many research groups have made concerted efforts to study the substrate-binding sites of MCR-1 and identify specific MCR-1 inhibitors. Through a series of painstaking efforts by many research groups, several versions of the high-resolution structures of cMCR-1 alone and in complex with two substrate analogs were obtained in a very short time. In addition, the enzymatic activity of this protein was tested in vitro and in vivo using these two substrate analogs, one of which was shown to have the function of MCR-1-inhibiting activity. These results have provided a potential drug prototype that could provide great theoretical guidance for the further design of MCR-1 inhibitors.

## Electronic supplementary material

ESM 1 (PDF 889 kb)
