# Supplementary material for: Recent progress on elucidating the molecular mechanism of plasmid-mediated colistin resistance and drug design
Source: Int Microbiol. 2019 Dec 23;23(3):355–66. doi: 10.1007/s10123-019-00112-1 (PMC7347692; doi:10.1007/s10123-019-00112-1)
Supplement: Supplementary file 1 — (PDF 889 kb) [file 10123_2019_112_MOESM1_ESM.pdf]

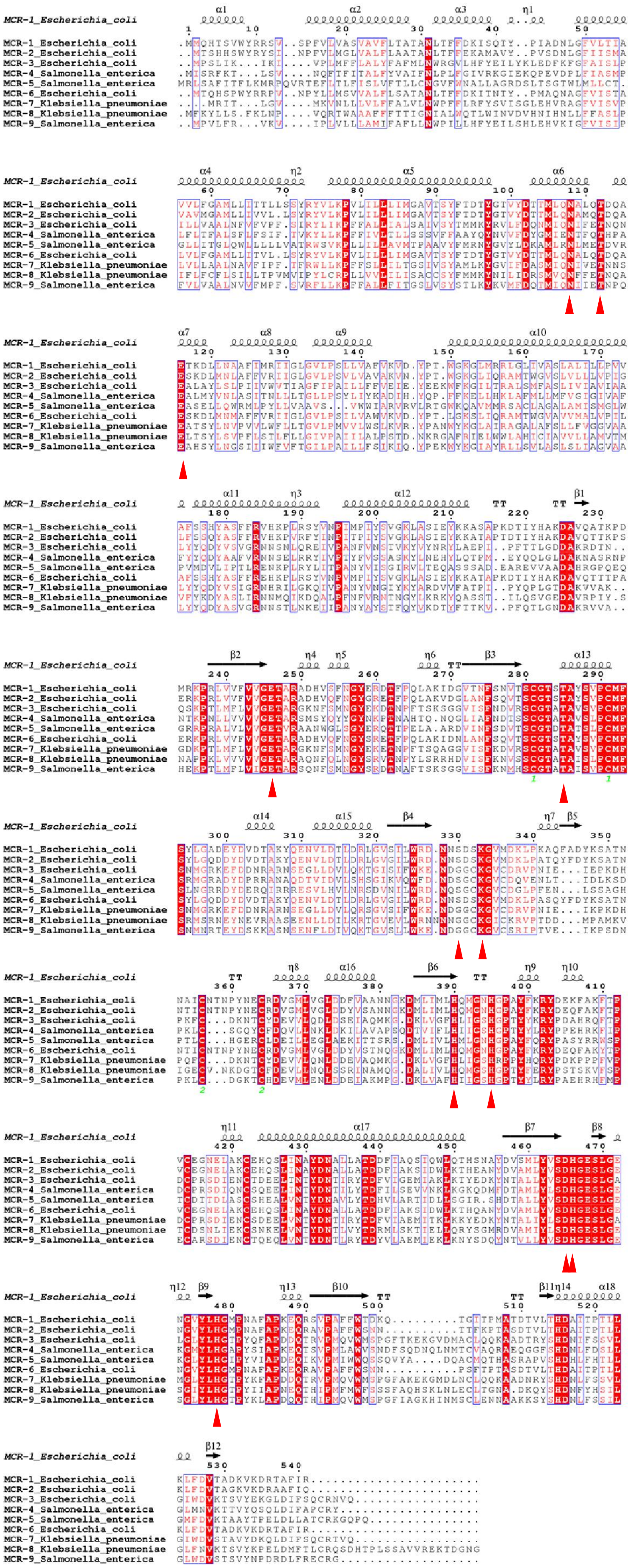

Supplementary Figure 1. Multiple sequence alignment of full-length MCR-1 and its variants. The important residues for catalyze reaction are highlighted by red triangles.
